# *Glycyrrhiza* extract alleviates hemorrhagic septicemia in *Triplophysa yarkandensis* infected with *Shewanella putrefaciens*: integrated bactericidal and immunomodulatory effects

**DOI:** 10.3389/fimmu.2026.1839757

**Published:** 2026-06-26

**Authors:** Huale Lu, Yu Tong, Wentao Zhu, Huijie Chen, Jianguo Su, Weimin Wang, Shouyi Zuo, Jie Wei, Zhaohua Huang, Zhulan Nie

**Affiliations:** 1 College of Life Science and Technology, Tarim University, Alar, China; 2 Xinjiang Production & Construction Corps Key Laboratory of Protection and Utilization of Biological Resources in Tarim Basin, Alar, China; 3 College of Animal Science and Technology Branch, Xinjiang Agricultural Vocational and Technical University, Changji, China; 4 College of Fisheries, Huazhong Agricultural University, Wuhan, China; 5 Alar Changxin Fisheries Co Ltd., Alar, China

**Keywords:** glycyrrhiza extract, immunomodulation, *Shewanella putrefaciens*, transcriptome, *Triplophysa yarkandensis*

## Abstract

**Background:**

Intensive saline-alkaline aquaculture has made *Shewanella putrefaciens* a lethal opportunistic pathogen threatening the indigenous economic fish *Triplophysa yarkandensis*, and antibiotic overuse poses severe ecological risks to this fragile ecosystem. The immune imbalance driving hemorrhagic septicemia progression and the therapeutic potential of herbal immunomodulators, such as *Glycyrrhiza* extract, in this species remain largely unexplored.

**Methods:**

We isolated and identified *S. putrefaciens* as the etiological agent of hemorrhagic septicemia in *T. yarkandensis*. Using a therapeutic model, we evaluated the efficacy of *Glycyrrhiza* extract via hematological/serum biochemical analyses, bacterial clearance assays, histopathology, head kidney transcriptomics, and qRT-PCR, alongside appropriate controls.

**Results:**

*Glycyrrhiza* extract significantly improved the survival of infected fish (58.1% vs. 43.3% in the PBS group), corrected abnormal physiological indices, accelerated bacterial clearance, relieved visceral inflammatory edema, and maintained respiratory and intestinal mucosal integrity. Transcriptomics revealed that it reversed infection-induced transcriptional chaos, with immune signaling and arachidonic acid metabolism as the core regulatory axes. qRT-PCR confirmed that it orchestrated pro-resolution immune responses via rapid acute inflammation activation and sustained anti-inflammatory cytokine upregulation.

**Conclusion:**

*Glycyrrhiza* extract acts as both a bacteriostat and a potent immunomodulator, resolving cytokine storm-induced immunopathology through integrated immune regulation. This offers a sustainable and eco-friendly strategy for managing bacterial diseases in extreme saline-alkaline aquatic environments, supporting its potential as a natural antibiotic alternative in aquaculture.

## 1 Introduction

The Yarkand loach (*Triplophysa yarkandensis*) is an economically and ecologically vital indigenous fish species endemic to the Xinjiang Uygur Autonomous Region of Northwest China (1). Exclusively distributed in the Tarim River Basin, this species has evolved remarkable physiological adaptations to survive in extreme aquatic environments characterized by high salinity, alkalinity and low temperatures (2). Owing to its robust resilience and distinct nutritional value, *T. yarkandensis* has recently become a focal species for aquaculture diversification in saline-alkaline regions (3). However, the transition from wild habitats to intensive farming has exposed this unique scaleless benthic fish to severe health challenges. The synergistic pressure of environmental stress (osmoregulatory burden in saline water) and high stocking density often compromises the mucosal immune barrier, rendering fish highly susceptible to opportunistic pathogens (4). Among these, hemorrhagic septicemia has emerged as a primary cause of mass mortality. However, the specific etiological agents and corresponding therapeutic strategies for this species remain largely unexplored.

While *Aeromonas* species are commonly associated with hemorrhagic diseases, *Shewanella putrefaciens*, a psychrotolerant (cold-adapted) specific spoilage organism, is emerging as a significant pathogen in freshwater aquaculture (5, 6). Given the low-temperature tolerance of *T. yarkandensis*, *S. putrefaciens* represents a particular threat, as its virulence factors are often upregulated at lower water temperatures, where other pathogens might be dormant (7). Clinically, *S. putrefaciens* infections manifest as severe ulcerative syndrome and systemic sepsis. Currently, the industry relies on broad-spectrum antibiotics, such as florfenicol, to control such outbreaks. However, in the fragile saline-alkaline ecosystems of the Tarim Basin, the indiscriminate use of antibiotics poses severe risks, including the rapid selection of multi-drug resistant (MDR) bacteria and disruption of the fish gut microbiota, which is critical for nutrient absorption in extreme environments (8). Consequently, there is an urgent demand for “green” therapeutic agents that can bolster the host immune response without causing environmental toxicity.

*Glycyrrhiza* (Licorice), derived from the roots of *Glycyrrhiza uralensis*, is a staple in traditional pharmacology and is widely cultivated in the arid, saline-alkaline soils of Northwest China—the same geographical region as *T. yarkandensis* (9). This presents a unique opportunity for a “locally sourced” therapeutic solution (10). *Glycyrrhiza* is rich in bioactive triterpenoid saponins (e.g., glycyrrhizin) and flavonoids, which possess potent anti-inflammatory and antiviral properties in mammalian models (11). In aquaculture, *Glycyrrhiza* has been shown to enhance non-specific immunity (e.g., such as lysozyme and complement activity) (12). However, most studies have focused on prophylaxis (prevention) (13, 14). A critical knowledge gap exists regarding its therapeutic efficacy, specifically whether *Glycyrrhiza* extract can actively resolve an established bacterial infection? Furthermore, does it act by directly killing bacteria or by modulating the “cytokine storm” to prevent immunopathological tissue damage, which is a crucial consideration for scaleless fish where skin integrity is paramount.

To bridge the gap between traditional herbal medicine and modern molecular immunology, this study integrated transcriptome analysis with quantitative pathology. We first isolated and identified a virulent strain of *S. putrefaciens* (LHL-1) from diseased *T. yarkandensis*, which satisfied Koch’s postulates. We established a clinical treatment model to compare the efficacy of *Glycyrrhiza* extract and florfenicol (15, 16). Uniquely, this study combined transcriptomics (RNA-seq), immune gene regulation (qRT-PCR), and quantitative histopathology (tissue edema indices) to decipher the therapeutic mechanism. We hypothesized that *Glycyrrhiza* extract confers protection by modulating the TLR/*NF-κB1* signaling pathway and arachidonic acid metabolism, thereby resolving systemic inflammation and maintaining mucosal barrier integrity (16, 17). This study provides the first mechanistic evidence for the use of plant-derived immunomodulators to treat emerging bacterial infections in indigenous fish species adapted to extreme saline-alkaline environments (18, 19).

## 2 Materials and methods

### 2.1 Experimental fish and ethical statement

Healthy Yarkand loach (*T. yarkandensis*), with an average weight of 9 ± 2 g, were sourced from Changxin Fishery (Alar, Xinjiang, China). Before the experiment, the fish were acclimated for two weeks in a recirculating saline-alkaline aquaculture system at 18 ± 1 °C and fed a commercial pellet diet at 2% body weight twice daily. All animal procedures were approved by the Animal Ethics Committee of Tarim University (approval no. PB20251231001) and conducted in strict accordance with the Guidelines for the Care and Use of Laboratory Animals.

### 2.2 Isolation and purification of the pathogenic bacterium

Fish exhibiting typical clinical signs of hemorrhagic septicemia, gill rot, and skin ulceration were collected from a local farm. Surface disinfection was performed using 70% ethanol. Necrotic muscle tissues were aseptically sampled and streaked on Luria-Bertani (LB) agar plates. After incubation at 28°C for 48 h, dominant single colonies exhibiting characteristic yellow pigmentation were selected and purified by streaking three times. The purified strain, designated LHL-1, was preserved in 50% glycerol at -80°C.

### 2.3 Pathogen identification

Colony morphology was recorded after 24 h of culture. Cellular morphology was examined by Gram staining using an optical microscope (PA53, Motic, Xiamen China). The ultrastructure, specifically the presence of surface appendages (pili/nanowires), was visualized using scanning electron microscopy (SEM, GeminiSEM 300, Zeiss, Germany) at an acceleration voltage of 10 kV. Physiological and biochemical characteristics were determined using microbial identification tubes (Hangzhou Microbial Reagent Co., Ltd., China), specifically assessing ornithine/lysine decarboxylase activity and sugar fermentation profiles consistent with those of *Shewanella* species.

Genomic DNA was extracted from the strain LHL-1. The 16S rRNA gene (primers 27F/1492R) and gyrB gene (primers GyrB-F/GyrB-R) were amplified, sequenced, and aligned using the NCBI BLAST. Phylogenetic trees were constructed using the neighbor-joining method in MEGA-X software (1,000 bootstrap replicates) to confirm species identity.

### 2.4 Antimicrobial susceptibility testing

The susceptibility of strain LHL-1 to 15 common veterinary antibiotics (including Florfenicol, Enrofloxacin, Cefotaxime, Ofloxacin, Ciprofloxacin, Ceftriaxone, Doxycycline, Oxytetracycline, Tetracycline, Norfloxacin, Amoxicillin, Co-trimoxazole, Penicillin, Neomycin and Erythromycin) was evaluated using the Kirby-Bauer disk diffusion method according to CLSI standards. Inhibition zone diameters (mm) were measured after 24 h of incubation at 28°C to screen for the most effective positive control drug.

### 2.5 LD_50_ determination and therapeutic efficacy

The strain LHL-1 was suspended in sterile PBS and serially diluted (1×10^5^ to 1×10^8^ CFU/mL). Healthy *T. yarkandensis* were randomly divided into 24 groups (combinations of injection volumes and concentrations). Mortality was recorded daily for 7 days to calculate the Median Lethal Dose (LD_50_) using the Reed-Muench method. The determined LD_50_ corresponded to a bacterial concentration of 5×10^6^ CFU/mL with an injection volume of 40 μL.

A blank control group (Control, n = 30) was established using completely healthy fish without any treatment or injection to provide physiological and immunological baselines. The remaining fish were intraperitoneally injected with the calculated LD_50_ dose of LHL-1. At 2 h post-infection (hpi), fish were randomly divided into three treatment groups (n = 30/group): phosphate-buffered saline (PBS) group, injected with 40 μL sterile PBS; *Glycyrrhiza* extract (GL) group, injected with 40 μL *Glycyrrhiza* extract; and florfenicol (FFC) group, injected with 40 μL florfenicol (2.5 mg/mL) (20).

The GL was prepared using a water extraction–alcohol precipitation method. Briefly, dried roots of *G. uralensis* were pulverized and extracted with distilled water at 80–90 °C for 2 h. The extract was filtered and concentrated, followed by ethanol precipitation to a final concentration of approximately 70%. The resulting extract was collected, evaporated, and reconstituted in sterile PBS to a final concentration of 20 mg/mL based on the dry weight of the crude extract (21). The extraction yield was approximately 8.3% (w/w, n = 3). This method has been applied in previous studies and is known to enrich triterpenoid saponins (e.g., glycyrrhizic acid) and flavonoids (11, 21, 22).

Cumulative mortality was recorded daily for seven days. To assess bacterial clearance, the liver, spleen, head kidney, trunk kidney, and muscle were collected from three fish per group on 1, 3, 5, and 7 days post-infection (dpi). Tissues were homogenized in sterile PBS, serially diluted, and plated on LB agar to quantify colony-forming units (CFU/g).

### 2.6 Serum biochemical and hematological analysis

At 1, 3, and 7 days post-infection (dpi), five fish per group were randomly selected for blood sampling. Blood samples (0.5 mL) were collected from the caudal vein using heparinized syringes. A 0.1 mL aliquot of each sample was used for hematological analysis, and the remaining sample was centrifuged at 3000 rpm for 10 min at 4 °C to separate the serum, which was stored at -80 °C for subsequent biochemical detection.

For hematological analysis, blood smears were prepared by spreading 10 μL of whole blood evenly on glass slides using the push-slide method, followed by air-drying and staining with Giemsa solution for 30 min. Stained smears were observed under a light microscope (400× magnification), with five random fields counted per slide to determine the percentage of red blood cells (RBC), white blood cells (WBC), and thrombocytes. For white blood cell subset analysis, 100 WBCs were counted per field to calculate the percentage of lymphocytes, neutrophils, and monocytes, with the results averaged across three replicate smears per fish.

### 2.7 Assessment of tissue edema and histopathology

At 7 dpi, the liver, head kidney, trunk kidney, and spleen were dissected, weighed (wet weight), and dried at 60 °C to constant weight (dry weight) to calculate the Wet/Dry ratio as an index of inflammatory edema.

For histopathological analysis, gill and hindgut tissues were fixed in 4% paraformaldehyde, embedded in paraffin, sectioned (4 μm), and stained with hematoxylin and eosin (H&E) to observe structural integrity, lamellar fusion, and mucosal damage.

### 2.8 RNA-seq and qRT-PCR validation

On 7 dpi, head kidney tissues (n=3/group) were collected and flash-frozen. Total RNA was extracted (Trizol), and the quality was verified (RIN ≥ 8.0). Sequencing libraries were constructed and sequenced using an Illumina NovaSeq PE150 platform. Differentially Expressed Genes (DEGs) were identified using DESeq2 (Padj< 0.05, ∣log2FoldChange∣>1). Functional enrichment analysis (GO and KEGG) was performed using clusterProfiler.

The six immune genes (*IL-1β*, *TNF-α*, *NF-κB1*, *IL-10*, *C3*, *IL-2*) were selected as core nodes of the TLR/*IL-10* axis with significant differential expression, covering inflammatory activation, signal transduction, inflammation resolution, innate and adaptive immunity for comprehensive validation. Specific primers for these genes and the reference gene *β-actin* were designed (**Table 1**). Prior to expression analysis, the amplification efficiency (*E*) of each primer pair was rigorously evaluated using standard curves generated from a 5-fold serial dilution of cDNA templates. All primers exhibited high amplification efficiencies ranging from 95.1% to 99.3%, which satisfies the theoretical assumptions required for relative quantification. Total RNA from Head Kidney and Spleen was reverse-transcribed, and qRT-PCR was performed on a CFX96 system using SYBR Green. Relative expression was calculated using the 2¯ΔΔCt method for all genes.

**Table 1:** Primer sequences used in qRT-PCR.

Gene name	Gene full name	Transcriptome ID	Primer name	Seq(5’→3’)	Amplification efficiency (E)
IL-1β	Interleukin-1 beta	TRINITY_DN962_c0_g1	IL1-β (F)	TCACCGAGTTCCAGCATGAG	96.4%
			IL1-β (R)	AGACACGCCGATCTCTTTCC	
TNF-α	Tumor necrosis factor alpha	TRINITY_DN85671_c0_g1	TNF-α (F)	TGGAAAGCGGTCTGGAGTCT	98.2%
			TNF-α (R)	AAGACAACCAGGATCAGGCA	
NF-κB1	Nuclear factor kappa B subunit 1	TRINITY_DN14633_c0_g1	NF-κB1 (F)	GCTGAAGGGAAGGTCGTGAA	97.1%
			NF-κB1 (R)	GTCTGATCGAGAGGTCAGCG	
C3	Complement component 3	TRINITY_DN98748_c0_g1	C3 (F)	TCTCGGGTGGCGTTTAGTTC	95.8%
			C3 (R)	TCCATTCGACCCAAAGCGAA	
IL-2	Interleukin-2	TRINITY_DN3239_c0_g1	IL-2 (F)	GAGTGTCCTTTCTCGCCTCC	95.1%
			IL-2 (R)	GACTGAGCAGGGAAACCCAA	
IL-10	Interleukin-10	TRINITY_DN4837_c1_g1	IL-10 (F)	ACAGCAAATCAAGCTCTCCCA	99.3%
			IL-10 (R)	AGGGTTTACCTTTCTTGCCACA	
β-actin	Beta-actin	TRINITY_DN118_c0_g1	β-actin (F)	GTAGAACTTCGGGGCTGCTT	97.6%
			β-actin (R)	CTGGCCCCTAGCACAATGAA	

### 2.9 Statistical analysis

Data are expressed as mean ± standard deviation (SD) (n=3). Survival analysis was performed using the log-rank (Mantel-Cox) test. Other data were analyzed using One-way ANOVA followed by Student’s t-test (GraphPad Prism 10.1.2). Significance was defined as *P < 0.05, **P < 0.01, and ***P < 0.001.

## 3 Results

### 3.1 Isolation, multi-dimensional identification and pathogenicity verification of the pathogen

**Figure 1:**
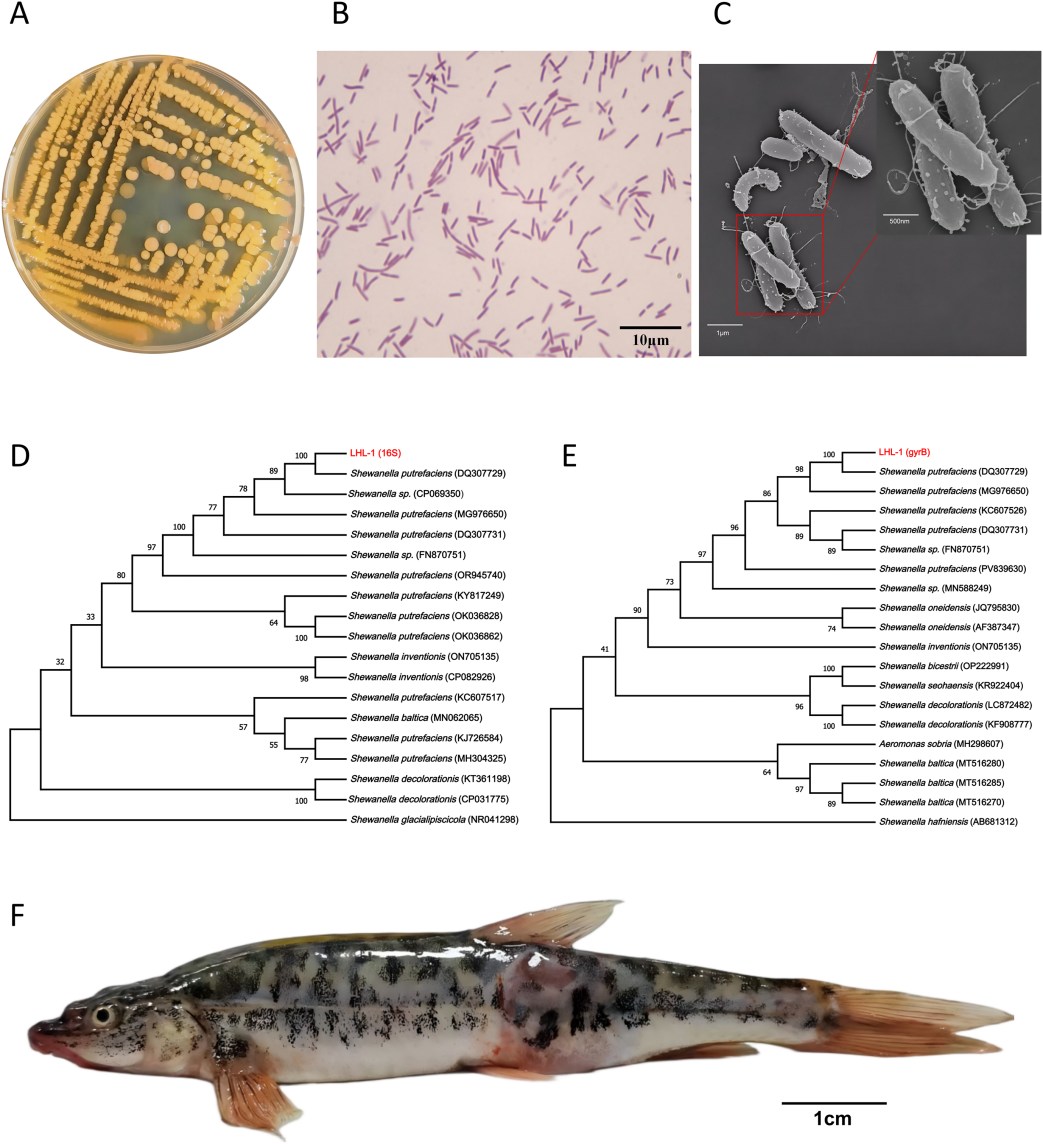
Identification and pathogenicity verification of *S. putrefaciens* strain LHL-1. **(A)** Colony morphology of strain LHL-1 on LB agar (48 h, 28°C), showing characteristic yellow pigmentation. **(B)** Gram-stained light microscopy revealing Gram-negative bacilli. Scale bar = 10 μm. **(C)** SEM observation of bacterial ultrastructure. Red box and inset show filamentous surface appendages (pili/nanowires) connecting cells. Scale bars: main = 1 μm; inset = 500 nm. **(D)** Neighbor-joining phylogenetic tree based on 16S rRNA gene sequences. Node values represent bootstrap support (1000 replicates). Strain LHL-1 (red) clusters with *S. putrefaciens*. **(E)** Neighbor-joining phylogenetic tree based on gyrB gene sequences, confirming species identity with higher resolution. **(F)** Clinical signs of *T. yarkandensis* in challenge model: severe dorsal ulceration and hemorrhaging consistent with natural infection.

A dominant bacterial strain, designated LHL-1, was isolated from the necrotic lesions of naturally diseased *T. yarkandensis*. It was identified as the etiological agent of hemorrhagic septicemia via morphological, physiobiochemical, and molecular biological analyses combined with pathogenicity verification (**Figure 1**, **Table 2**). Morphological characterization revealed that the strain formed circular, smooth colonies with a yellow-orange pigment on LB agar after 24 h of incubation (**Figure 1A**) and was identified as gram-negative short bacilli (**Figure 1B**). SEM observations showed that the bacterial cells were 1.5-2.0 μm in length and possessed surface filamentous appendages resembling pili/nanowires that connected adjacent cells (**Figure 1C**), suggesting strong adhesive and biofilm-forming capabilities. The physiobiochemical profile was characterized by positive reactions for ornithine decarboxylase and negative reactions for lysine decarboxylase, along with the ability to ferment glucose and other carbohydrates (**Table 2**), which was highly consistent with the phenotypic traits of *S. putrefaciens*. NCBI BLAST alignment revealed that both the 16S rRNA and gyrB gene sequences of strain LHL-1 shared 99.93% sequence identity with the reference strains of *S. putrefaciens*. Moreover, phylogenetic trees constructed based on these sequences both demonstrated that LHL-1 clustered with the reference strains of *S. putrefaciens* with 100% bootstrap support (**Figures 1D, E**), confirming its species identity at the molecular level. Artificial challenge tests fulfilled Koch’s postulates: healthy *T. yarkandensis* injected with LHL-1 exhibited 100% morbidity within 7 days and developed typical clinical signs consistent with natural infection, including dorsal ulceration, hemorrhaging at the fin bases, and muscle necrosis (**Figure 1F**). The same strain was re-isolated from the visceral organs of the challenged fish, confirming that LHL-1 is the pathogenic *S. putrefaciens* strain causing hemorrhagic septicemia in *T. yarkandensis*.

**Table 2:** Physiological and biochemical characteristics of strain LHL-1.

Identification item	Result	Identification item	Result
L-Aspartate Aminase	+	D-Fructose	+
α-Glucosidase	+	Glucose	+
Ornithine Decarboxylase	+	Lactose	+
Catalase	+	Maltose	+
Urease	–	D-Mannitol	–
β-Galactosidase	–	D-Xylose	–
Lysine Decarboxylase	–	Malic Acid	+
β-Glucosidase	–	Methyl Red Test	–

“+” indicates a positive reaction; “-” indicates a negative reaction.

### 3.2 *In vitro* antibiotic susceptibility profile of the pathogenic strain

To screen for a positive control drug for *in vivo* therapeutic experiments, the *in vitro* susceptibility of LHL-1 to 15 common veterinary antibiotics was determined using the Kirby-Bauer disk diffusion method (**Table 3**). The results showed that the strain exhibited broad-spectrum susceptibility to most tested antibiotics, with inhibition zone diameters ranging from 17.41 to 44.57 mm. Florfenicol had the strongest bacteriostatic activity, with an inhibition zone diameter of 44.57 ± 0.48 mm, followed by enrofloxacin and cefotaxime. The strain was highly sensitive to fluoroquinolones and tetracyclines, whereas it showed significantly lower susceptibility to macrolides (erythromycin) and aminoglycosides (neomycin), which are the two classes of antibiotics with the weakest *in vitro* bacteriostatic effects. Notably, florfenicol is a legally approved first-line broad-spectrum antibiotic commonly used to treat gram-negative bacterial septicemia in the saline-alkaline aquaculture of *T. yarkandensis* and other loach species in China. It also exhibits excellent tissue penetration, high stability in high-salt and high-alkaline environments, and standardized dosage regimens. Based on the above results, florfenicol with optimal bacteriostatic activity was selected as the positive control drug for subsequent *in vivo* therapeutic experiments, establishing a reliable benchmark for evaluating the therapeutic efficacy of *Glycyrrhiza* extract.

**Table 3:** Antimicrobial susceptibility profile of *S. putrefaciens* strain LHL-1.

Antimicrobial agent	Inhibition zone diameter (mm)	Antimicrobial agent	Inhibition zone diameter (mm)
Florfenicol	44.57 ± 0.48	Tetracycline	35.12 ± 0.36
Enrofloxacin	43.68 ± 0.13	Norfloxacin	32.15 ± 2.74
Cefotaxime	43.15 ± 0.22	Amoxicillin	29.44 ± 1.35
Ofloxacin	41.40 ± 0.04	Co-trimoxazole	29.10 ± 1.23
Ciprofloxacin	39.44 ± 0.32	Penicillin	24.75 ± 0.17
Ceftriaxone	37.19 ± 1.68	Neomycin	18.03 ± 3.12
Doxycycline	36.85 ± 0.34	Erythromycin	17.41 ± 2.05
Oxytetracycline	36.68 ± 2.08		

Data are presented as mean ± standard deviation (SD) of three independent replicates.

### 3.3 Survival protection and tissue bacterial clearance efficacy of *Glycyrrhiza* extract in infected fish

**Figure 2:**
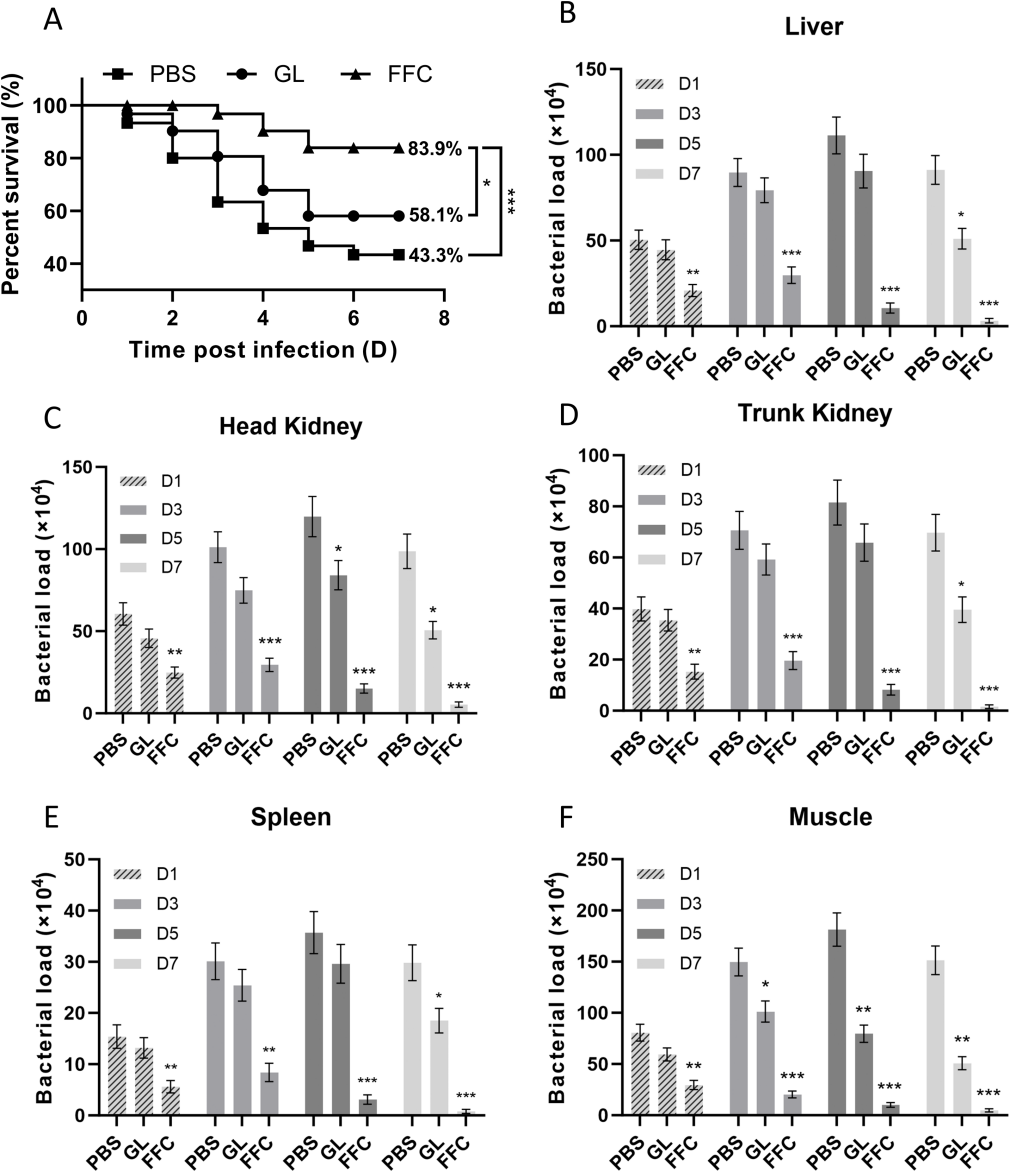
Therapeutic efficacy of *Glycyrrhiza* extract against *S. putrefaciens* infection in *T. yarkandensis*. **(A)** Kaplan-Meier cumulative survival curves of *T. yarkandensis* over a 7-day period following *S. putrefaciens* challenge and subsequent treatment with PBS, GL, or FFC. Significance was determined using the Log-rank (Mantel-Cox) test. **(B–F)** quantification of bacterial loads in the **(B)** liver, **(C)** head kidney, **(D)** trunk kidney, **(E)** spleen, and **(F)** muscle at 1, 3, 5, and 7 days post-infection. Data are presented as mean ± standard deviation (SD) (n=3). Asterisks indicate statistically significant differences compared to the PBS control group at the same time point (*P < 0.05, **P < 0.01, ***P < 0.001).

A therapeutic challenge model of *S. putrefaciens* infection in *T. yarkandensis* was established to evaluate the *in vivo* core therapeutic efficacy of *Glycyrrhiza* extract, which significantly improved the survival rate of infected fish and promoted tissue bacterial clearance (**Figure 2**). Survival analysis showed that the PBS group suffered acute mortality starting at 1 d post-infection (dpi), with a cumulative survival rate of only 43.3% at 7 dpi. The GL group significantly delayed the onset of mortality, with the cumulative survival rate increasing to 58.1% at 7 dpi (*P* < 0.05), representing a 14.8% absolute increase in protection rate compared with the PBS group. The FFC-positive control group had the highest survival rate of 83.9% (*P* < 0.001) (**Figure 2A**). The determination of tissue bacterial clearance kinetics indicated that the bacterial load in the liver, head kidney, posterior kidney, spleen and muscle of the PBS group increased in a time-dependent manner and peaked at 5 dpi, showing characteristics of systemic septicemia. The GL group exhibited significant bacteriostatic effects in all tested tissues, with the bacterial load at 7 dpi being significantly lower than that in the PBS group (*P* < 0.05 or *P* < 0.01). In contrast, the FFC group achieved rapid and near-complete bacterial clearance at 7 dpi (**Figures 2B–F**).

### 3.4 *Glycyrrhiza* extract ameliorates hematological and serum biochemical disorders in infected *T. yarkandensis*

**Figure 3:**
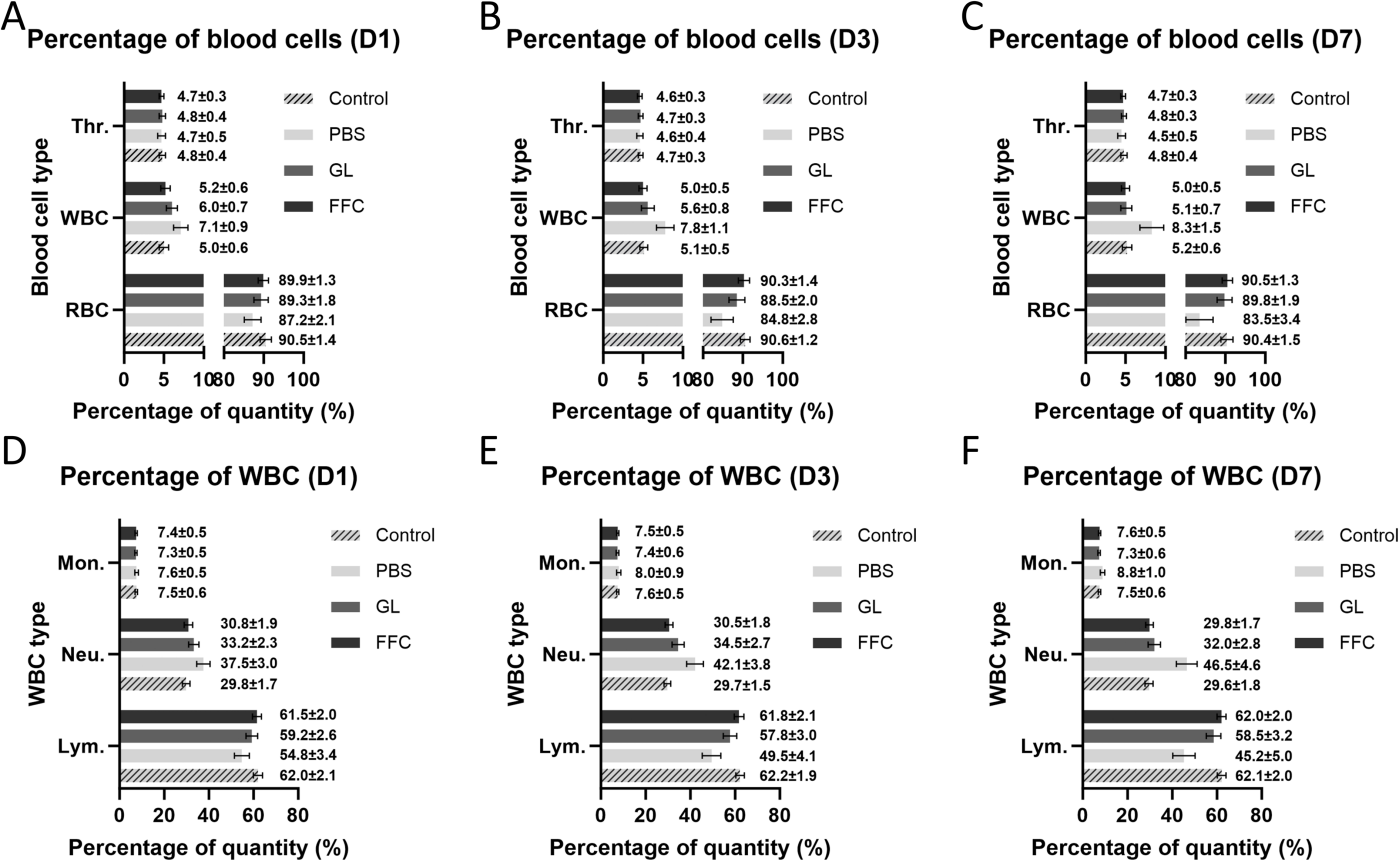
Changes in hematological parameters and leukocyte subpopulations of *T. yarkandensis* infected with *S. putrefaciens* at 1, 3, and 7 days post-infection (dpi). **(A–C)** proportions of thrombocytes (Thr.), WBC, and RBC in peripheral blood at 1 dpi **(A)**, 3 dpi **(B)**, and 7 dpi **(C)**. **(D–F)** proportions of monocytes (Mon.), neutrophils (Neu.), and lymphocytes (Lym.) in leukocytes at 1 dpi **(D)**, 3 dpi **(E)**, and 7 dpi **(F)**.

Hematological parameters at 1, 3, and 7 days post-infection (dpi) revealed that *S. putrefaciens* infection severely disrupted peripheral blood cell homeostasis in the PBS group (**Figure 3**). The percentage of RBC was continuously reduced, while the WBC proportion was showed a continuous increase from D1 to D7 in the PBS group, with no significant differences in thrombocyte percentage were observed among all groups (**Figures 3A–C**). For leukocyte subsets (**Figures 3D–F**), the PBS group exhibited persistent increases in neutrophil and monocyte percentages, coupled with a striking decrease in lymphocyte percentage, indicating excessive inflammation and immune exhaustion. GL treatment markedly ameliorated these abnormalities by increasing RBC and lymphocyte proportions and decreasing WBC, neutrophil, and monocyte percentages at all time points, and the FFC group maintained hematological indicators comparable to the healthy control group.

**Figure 4:**
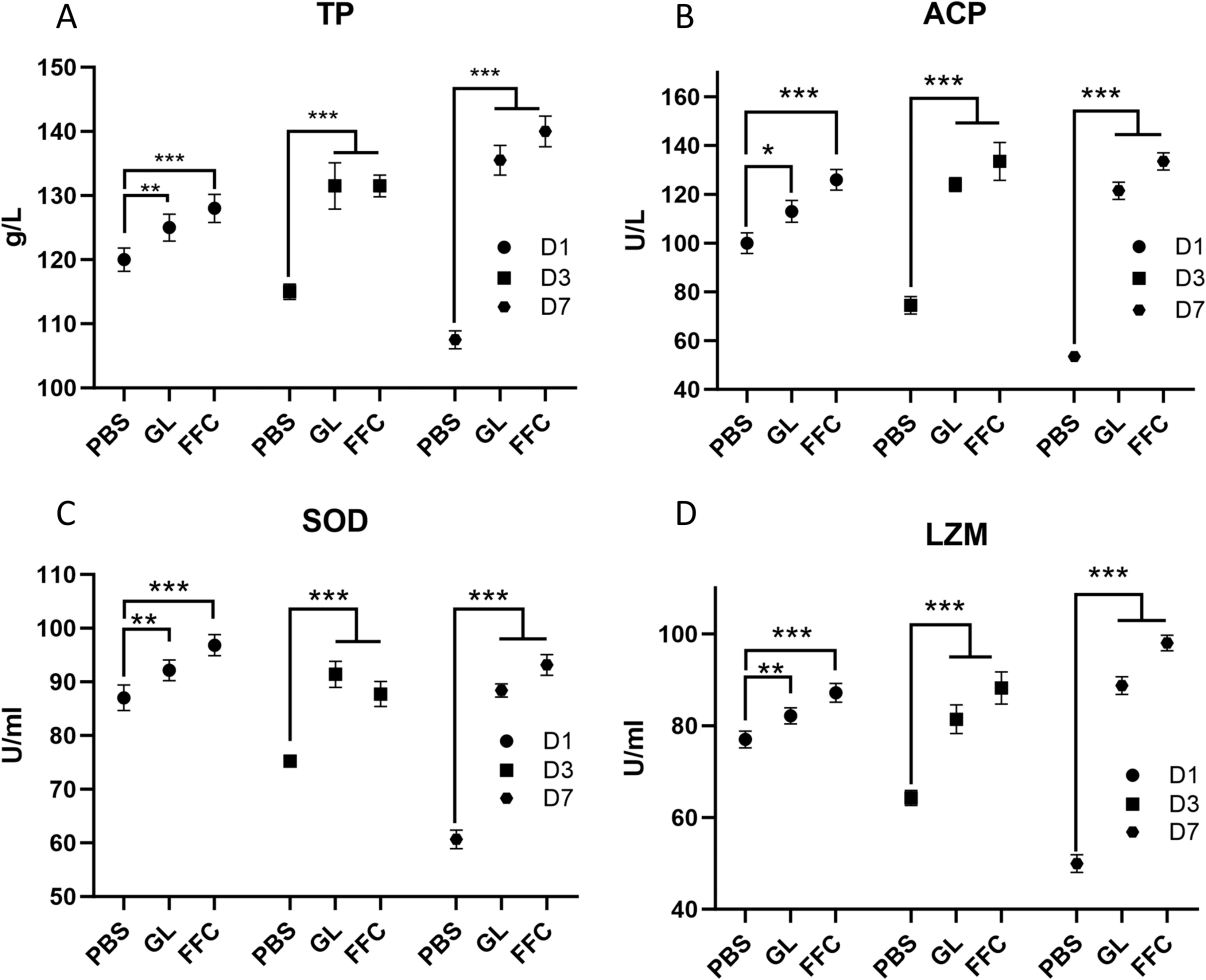
Modulation of serum biochemical and immunological parameters in *T. yarkandensis* following *S. putrefaciens* infection and treatment. Changes in **(A)** TP content, **(B)** ACP activity, **(C)** SOD activity, and **(D)** LZM activity in serum at 1, 3, and 7 days post-infection. Data represent mean ± standard deviation (n=3). Statistical significance compared to the PBS group at the same time point is indicated by asterisks: *P < 0.05, **P < 0.01, ***P < 0.001.

Serum biochemical indices further verified the protective effects of *Glycyrrhiza* extract on immune and antioxidant function (**Figure 4**). In the PBS group, serum total protein (TP) content (**Figure 4A**), acid phosphatase (ACP) activity (**Figure 4B**), superoxide dismutase (SOD) activity (**Figure 4C**) and lysozyme (LZM) activity (**Figure 4D**) all declined continuously and reached their lowest levels at 7 dpi, reflecting metabolic disorder, immune failure, and severe oxidative stress. In contrast, GL treatment significantly elevated TP content and enhanced ACP, SOD, and LZM activities compared with the PBS group at 3 and 7 dpi. The FFC group maintained the highest levels of all serum indices, preserving complete physiological, immune, and antioxidant homeostasis.

### 3.5 Attenuation of tissue inflammatory edema and protection of mucosal barrier integrity by *Glycyrrhiza* extract in infected fish

**Figure 5:**
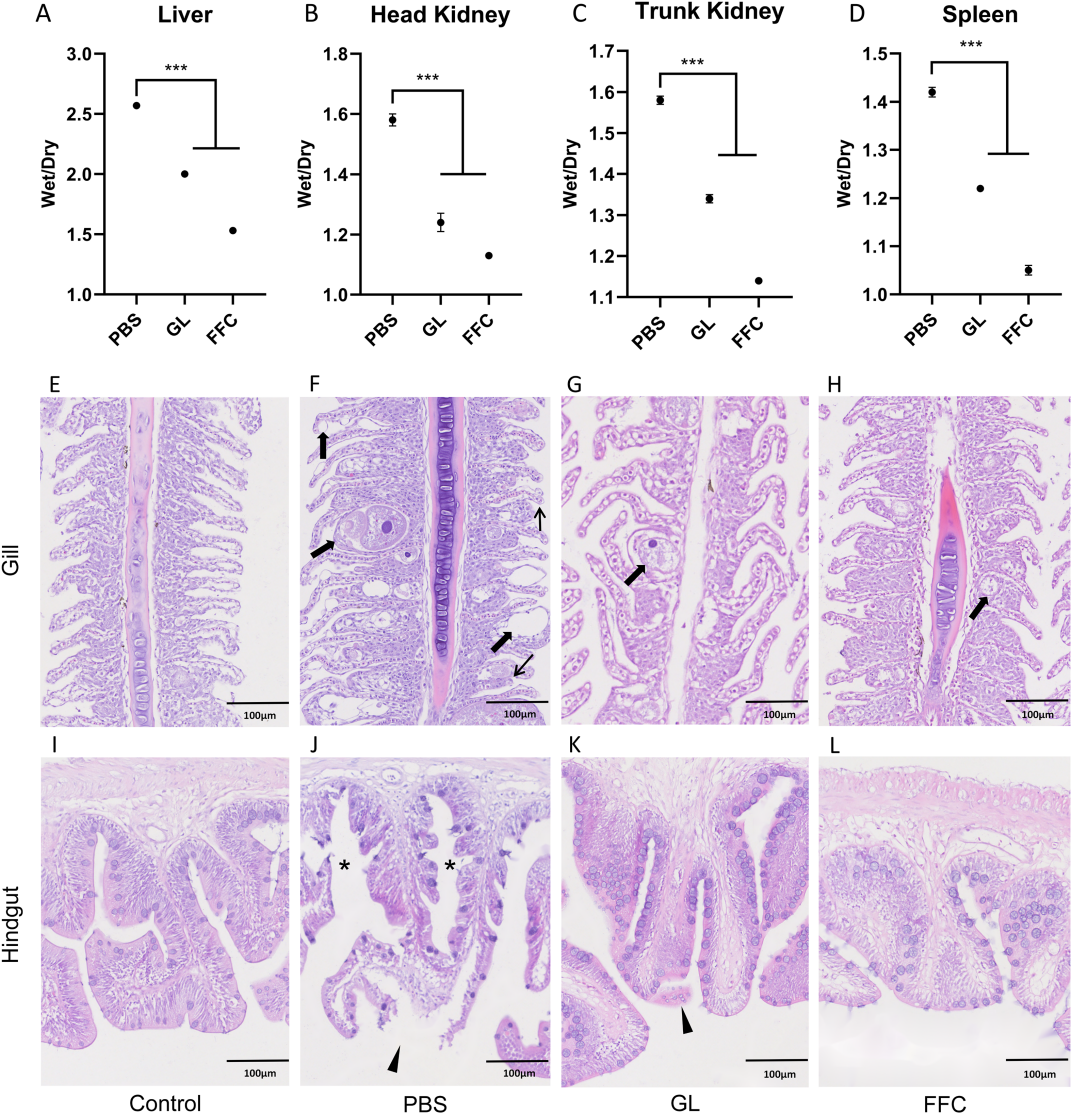
Evaluation of tissue edema and histopathological changes in *T. yarkandensis* following *S. putrefaciens* infection and treatment. **(A–D)** Quantitative assessment of tissue edema via wet/dry weight ratios in the **(A)** liver, **(B)** head kidney, **(C)** trunk kidney, and **(D)** spleen at 7 dpi. Higher ratios indicate severe inflammatory edema. Data represent mean ± SD. ***P < 0.001 indicates a significant reduction in edema in the GL and FFC groups compared to the PBS. **(E–H)** Representative histopathology of gill tissues (H&E staining, scale bar = 100 μm). **(E)** Control: Normal gill filament structure. **(F)** PBS: severe pathology characterized by lamellar fusion (thin arrows) and inflammatory cysts (thick arrows). **(G)** GL: alleviated damage with patent inter-lamellar spaces, despite occasional residual cysts (thick arrow). **(H)** FFC: restored structure with minimal inflammation (thick arrow). **(I–L)** representative histopathology of hindgut tissues (H&E staining, scale bar = 100 μm). **(I)** Control: intact villi with continuous epithelium. **(J)** PBS: bacterial enteritis showing severe villous atrophy (asterisks, *) and apical necrosis (arrowheads). **(K)** GL: preserved mucosal integrity; arrowhead indicates a restored villus tip. **(L)** FFC: restored intestinal morphology.

Using the Wet/Dry weight ratio as the quantitative index of inflammatory edema and combined with histopathological examination by hematoxylin and eosin (H&E) staining, it was confirmed at the tissue morphological level that *Glycyrrhiza* extract could significantly alleviate infection-induced systemic inflammatory injury and effectively protect the structural integrity of respiratory and intestinal mucosal barriers (**Figure 5**). Tissue edema analysis showed that at 7 dpi, the liver, head kidney, trunk kidney, and spleen of the PBS group presented severe hydropic degeneration, with the liver having a wet/dry weight ratio exceeding 2.5, which was significantly higher than that of the FFC group (~1.5). The GL group significantly reduced the wet/dry weight ratios of the above four tissues (*P* < 0.001), restoring the organ hydration level to near the baseline of the antibiotic-treated group and effectively alleviating systemic inflammatory edema (**Figures 5A–D**). Mucosal barrier pathological observations revealed that the PBS group had severe gill epithelial hyperplasia and complete fusion of secondary lamellae, with a large number of inflammatory cysts in the gill filament structure and severely impaired respiratory surface area. The hindgut showed villous atrophy, widened inter-villous spaces, and apical epithelial necrosis, presenting classic signs of bacterial enteritis (**Figures 5F, J**). In contrast, the gill filament structure of the GL group was significantly recovered, with the secondary lamellae remaining largely distinct and unfused, with only a small number of residual inflammatory cysts, effectively preserving the respiratory surface area. The intestinal mucosal structure of the GL group was intact, with elongated and continuous villi and no necrosis at the villous tips, successfully maintaining the integrity of the intestinal-vascular barrier and preventing pathogen translocation and endotoxin entry into systemic circulation (**Figures 5G, K**). Its tissue morphology was similar to that of the healthy control (**Figures 5E, I**) and FFC groups (**Figures 5H, L**).

### 3.6 Transcriptomic analysis and verification of key immune genes for *Glycyrrhiza* extract-mediated regulation of immune homeostasis

**Figure 6:**
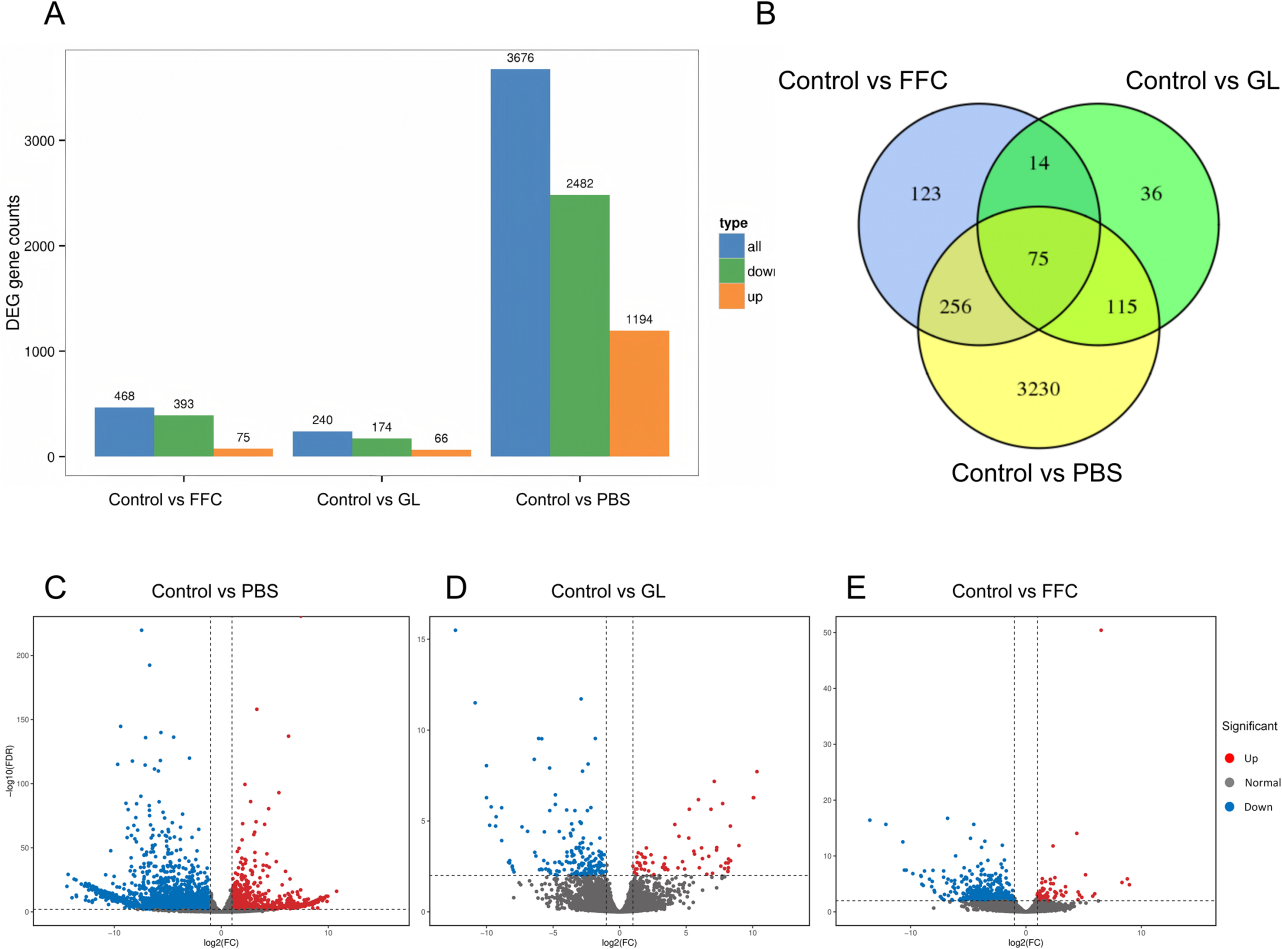
Overview of transcriptomic changes in the head kidney of *T. yarkandensis* following *S. putrefaciens* infection and treatment. **(A)** Bar chart showing the number of DEGs in each pairwise comparison against the healthy control (control). Blue bars: total DEGs; green bars: downregulated; orange bars: upregulated. Note the massive number of DEGs in the untreated PBS group versus the minimal DEGs in the GL group. **(B)** Venn diagram showing the overlap of DEGs among the three comparison groups (control vs PBS, control vs GL, control vs FFC). The large number of unique genes in the Control vs PBS circle (3230) represents the pathological gene signature resolved by treatment. **(C–E)** Volcano plots visualizing the distribution of DEGs in **(C)** Control vs PBS, **(D)** Control vs GL, and **(E)** Control vs FFC comparisons. Red dots indicate significantly upregulated genes, blue dots indicate downregulated genes, and grey dots indicate non-significant genes (∣log_2_FC∣>1, FDR<0.05).

**Figure 7:**
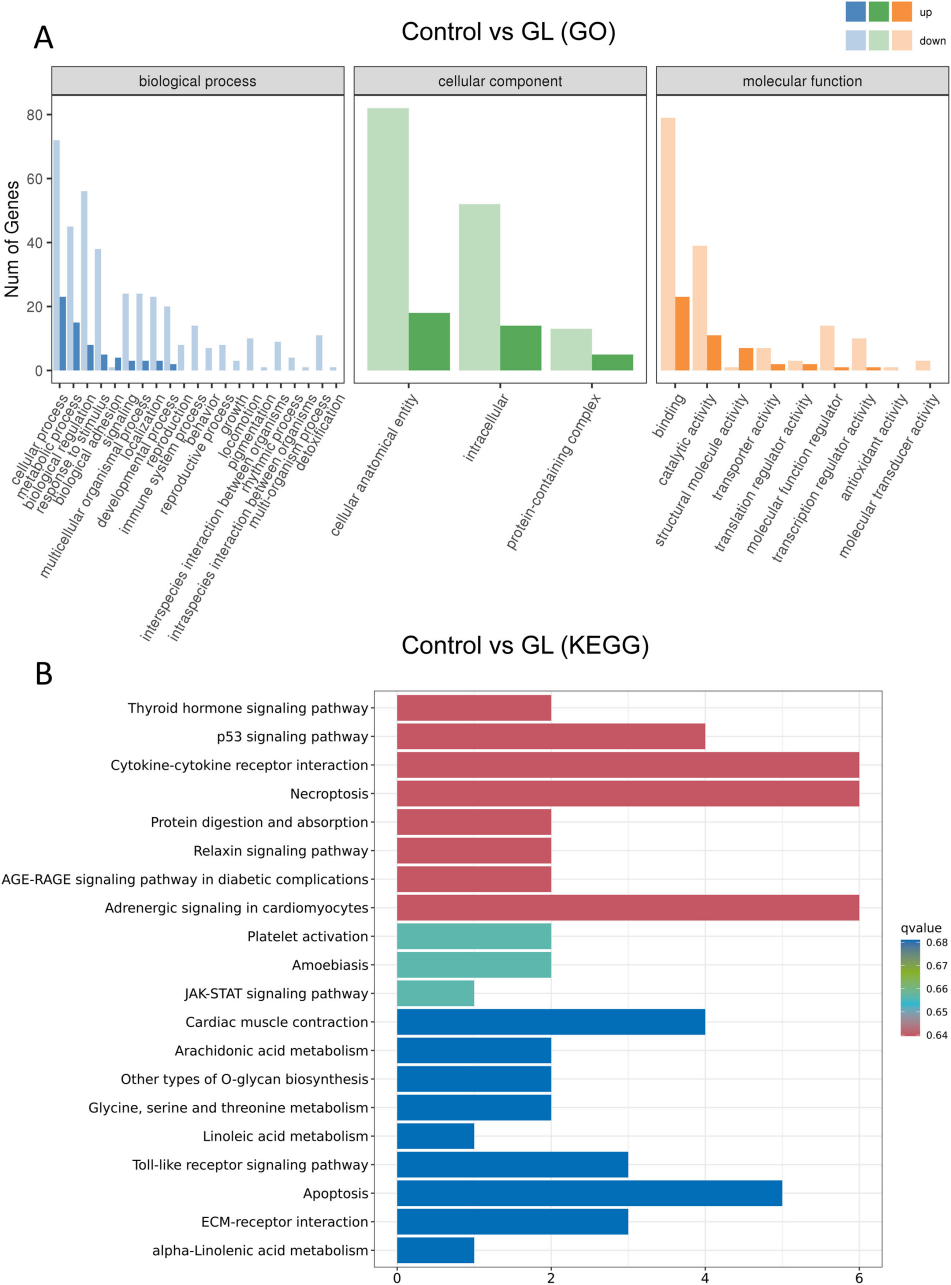
Functional enrichment analysis of DEGs in the *Glycyrrhiza*-treated group (control vs GL). **(A)** GO classification. Histograms display the number of up-regulated (dark color) and down-regulated (light color) genes assigned to biological process, cellular component, and molecular function. Note the low number of DEGs (y-axis scale), indicating a return to transcriptional homeostasis. **(B)** KEGG pathway enrichment analysis. Top 20 enriched signaling pathways based on Q-value (color scale) and gene count (bar height). The analysis highlights the modulation of “Toll-like receptor signaling,” “Apoptosis,” and “Arachidonic acid metabolism” as key regulatory mechanisms induced by *Glycyrrhiza* treatment.

**Figure 8:**
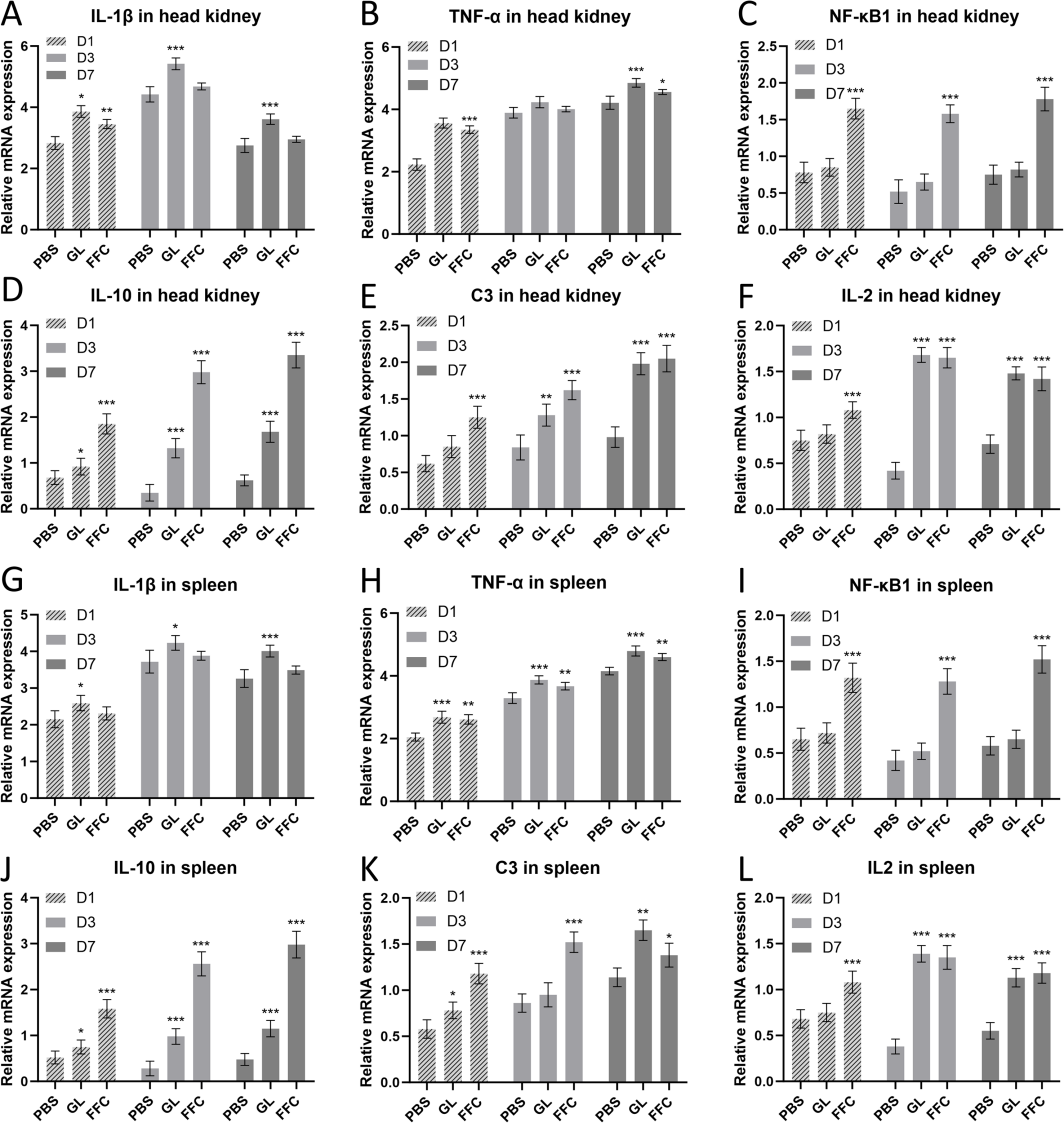
Temporal expression profiles of immune-related genes in *T. yarkandensis* following *S. putrefaciens* infection and treatment. Relative mRNA expression levels of **(A, G)** *IL-1β*, **(B, H)** *TNF-α*, **(C, I)** *NF-κB1*, **(D, J)** *IL-10*, **(E, K)** Complement component 3 (*C3*), and **(F, L)** *IL-2* in the head kidney (top panels) and spleen (bottom panels) at 1, 3, and 7 days post-infection. Expression levels were normalized to the housekeeping gene *β-actin* and calibrated against the D1 PBS group. Data represent mean ± SD (n=3). Asterisks indicate statistically significant differences compared to the PBS group at the same time point (*P < 0.05, **P < 0.01, ***P < 0.001). Note the significant upregulation of the anti-inflammatory cytokine *IL-10* in the GL and FFC groups compared to the PBS group.

Taking the head kidney, the central immune organ of fish, as the research object, RNA-seq combined with qRT-PCR verification was performed to elucidate the core mechanism of *Glycyrrhiza* extract in regulating immune homeostasis at the molecular level, confirming that it mediates the active resolution of inflammation and reshapes immune homeostasis via the TLR/*IL-10* axis (**Figures 6**–**8**; **Supplementary Figures 1**, **2**). Analysis of DEGs showed that 3,676 DEGs were identified between the healthy control group and the PBS-infected group, suggesting that *S. putrefaciens* infection induced severe disorder of host gene expression. In contrast, only 240 DEGs were detected between the healthy control group and the GL group, which was much fewer than the 468 DEGs between the healthy control group and the FFC group. Moreover, the PBS group had 3,230 unique DEGs that were not found in the treatment groups. Volcano plots also showed that the gene expression distribution of the GL group was much closer to that of the healthy control group, indicating that *Glycyrrhiza* extract can effectively reverse the transcriptional chaos induced by infection and restore the gene expression pattern to a baseline close to that of healthy fish (**Figures 6A–E**). GO and KEGG pathway enrichment analyses showed that the DEGs in the GL group were only enriched in fundamental biological processes, such as cellular and metabolic processes (**Figure 7A**), which was similar to the GO enrichment profile of the FFC group (**Supplementary Figure 1B**). In contrast, thousands of genes in the PBS group induced by *S. putrefaciens* infection were enriched in various biological processes (**Supplementary Figure 1A**), further confirming that *Glycyrrhiza* extract can effectively inhibit the systemic transcriptional disorder caused by the infection. The core regulatory pathways of the GL group were specifically enriched in the Toll-like receptor (TLR) signaling pathway, apoptosis, and arachidonic acid metabolism (**Figure 7B**), which were completely different from the broad “cytokine storm” KEGG enrichment profile of the PBS group (**Supplementary Figure 2A**) and essentially distinct from the survival pathways, such as MAPK and PI3K-Akt, enriched in the FFC group (**Supplementary Figure 2B**). This suggests that *Glycyrrhiza* extract does not merely eliminate pathogens but mediates the resolution of inflammation by regulating host immunity and lipid metabolism. Verification of the temporal expression of six key immune genes by qRT-PCR further confirmed that *Glycyrrhiza* extract significantly upregulated the expression of the pro-inflammatory cytokines *IL-1β* and *TNF-α* in the early stage of infection (*P* < 0.001), rapidly initiating the acute inflammatory response for bacterial clearance. Meanwhile, it induced a time-dependent significant upregulation of the anti-inflammatory cytokine *IL-10*, with the *IL-10* expression level in the spleen at 7 dpi being approximately 2.4-fold higher than that in the PBS group (*P* < 0.001), thus forming an inflammatory “brake” mechanism. In addition, the expression of *NF-κB1* (a key transcription factor of the TLR pathway), *C3* (a humoral immunity marker), and *IL-2* (an adaptive immunity marker) in the GL group was significantly upregulated at 3–7 dpi, which was highly consistent with the transcriptomic enrichment results (**Figures 8A–L**). These findings confirmed that *Glycyrrhiza* extract achieves precise immune regulation from the activation of acute inflammation to the active resolution of inflammation by regulating the TLR/*IL-10* axis, thereby reshaping the immune homeostasis of infected fish.

## 4 Discussion

The expansion of aquaculture into extreme environments, such as the saline-alkaline waters of the Tarim Basin, presents unique challenges. While *S. putrefaciens* is classically defined as a specific spoilage organism, this study confirmed its role as a lethal opportunistic pathogen in *T. yarkandensis*. The filamentous appendages (pili/nanowires) observed by SEM in *S. putrefaciens* LHL-1 (**Figure 1C**) (23) are important virulence-associated structures. These appendages facilitate bacterial adhesion to host mucosal surfaces and promote biofilm formation, thereby enhancing colonization on the gill and intestinal mucosa of *T. yarkandensis* and contributing to the progression of systemic septicemia (24, 25). Furthermore, the antibiotic susceptibility profile revealed that although LHL-1 remained sensitive to florfenicol, it exhibited intrinsic resistance to macrolides and aminoglycosides. This underscores the urgency of developing non-antibiotic therapies, as the reliance on single-class antibiotics in closed recirculating systems risks rapid selection of multi-drug resistance (26).

We agree that the magnitude of survival improvement is a key indicator of therapeutic efficacy. In this study, GL increased survival from 43.3% to 58.1% (an absolute improvement of 14.8%, *P* < 0.05). While this represents a statistically significant effect, the magnitude was moderate compared with antibiotics such as florfenicol. Importantly, the present study was based on a strict therapeutic model (treatment at 2 h post-infection) rather than a prophylactic model. Most reports demonstrating higher survival improvements (20–30% or more) have been conducted under prophylactic administration, where host immunity is pre-activated before pathogen challenge. Post-infection intervention is inherently more challenging and usually results in relatively lower survival gains. As a plant-derived immunomodulator, GL showed moderate but meaningful therapeutic efficacy in line with previous studies (18, 19, 27). Survival from septicemia depends not only on pathogen clearance but also on the control of excessive inflammatory responses. GL significantly improved hematological parameters, alleviated tissue edema, and restored transcriptomic homeostasis, which collectively contributed to host survival but may not be fully reflected by the survival rate alone. Since GL did not reach the survival efficacy of florfenicol (83.9%), it is more appropriately regarded as a complementary or adjunct strategy to reduce antibiotic reliance, rather than a direct substitute. The low risk of drug resistance and potent immunoregulatory properties make GL a sustainable option for integrated disease management in saline-alkaline aquaculture (16, 28).

From an application perspective, it is important to clearly distinguish between prophylactic and therapeutic strategies in aquaculture disease control. Prophylactic approaches, such as dietary supplementation with plant-derived immunostimulants, aim to enhance host resistance prior to pathogen exposure and often result in higher apparent survival rates. However, their effectiveness depends on accurate prediction of infection timing and may be limited under dynamic farming conditions. In contrast, the therapeutic strategy employed in this study, with intervention initiated after infection establishment, more closely reflects real-world aquaculture scenarios. Although this approach is inherently more challenging and typically yields lower survival improvements, it provides a more realistic evaluation of treatment efficacy. The present results indicate that *Glycyrrhiza* extract can confer measurable protection even under these stringent conditions. Nevertheless, its moderate survival benefit suggests that therapeutic use alone may be insufficient for acute infection control, and it may be more effective when integrated with prophylactic strategies or combined with antimicrobial agents.

While the antibiotic florfenicol achieved rapid bacterial clearance (**Figure 2**), the GL group exhibited significantly reduced yet still detectable bacterial loads across multiple tissues, particularly in the head kidney and muscle. This pattern indicates that GL functions primarily through immunomodulatory and bacteriostatic mechanisms rather than rapid bactericidal eradication. Notably, GL significantly reduced bacterial loads at 1 dpi in the head kidney and muscle (*P* < 0.05), indicating accelerated early-stage bacterial control. Concurrently, GL improved survival, alleviated inflammatory edema, and preserved mucosal barrier integrity, consistent with its role in orchestrating pro-resolution immune responses characterized by early activation of acute inflammation followed by sustained anti-inflammatory cytokine upregulation, as confirmed by transcriptomic and qRT-PCR analyses. The persistence of low-level bacterial loads in GL-treated fish is therefore more consistent with immune-mediated tolerance and controlled resolution of inflammation than with a chronic carrier state. Moreover, no obvious evidence of a chronic carrier state was observed in surviving fish. The early reduction in bacterial loads also suggests that GL components (likely glycyrrhizin) may interfere with bacterial membrane stability or disrupt adhesive pili structures identified by SEM, thereby limiting initial colonization (27, 29). Hematological analyses further validated the immunoregulatory effects of GL (**Figure 3**). Infection with S. putrefaciens caused a marked decline in RBC and a continuous rise in WBC in the PBS group, accompanied by increased neutrophils and monocytes and decreased lymphocytes, indicative of a severe inflammatory storm and adaptive immune collapse. In contrast, GL treatment effectively restored the proportions of RBC, WBC, and leukocyte subsets, suppressing excessive neutrophilic inflammation and reversing lymphopenia. This hematological homeostasis reflects the ability of GL to maintain physiological oxygen transport and restrain immunopathological damage, which is critical for the survival of fish with sepsis (30). However, survival in septicemia is often determined by the host’s ability to tolerate inflammation and not just eliminate the pathogen. The “cytokine storm” hypothesis suggests that excessive proinflammatory signaling leads to vascular leakage and organ failure (31, 32). Our data support this: the untreated PBS group suffered from massive hepatic and renal edema (**Figure 4A**), which correlated with a collapse in serum protein and antioxidant levels (**Figure 4**) (33). In contrast, GL treatment maintained the physiological homeostasis. The preservation of SOD activity and the reduction in tissue wet/dry ratios indicate that GL effectively scavenges reactive oxygen species (ROS) and protects vascular endothelium, thereby preventing the lethal edema associated with septic shock (34).

The integration of transcriptomics and qRT-PCR provides a sophisticated mechanistic explanation for the observed phenotypes. In the PBS group, the transcriptome was characterized by a massive dysregulation of metabolic and cytokine-receptor interaction pathways (**Figures 6**, **8**), reflecting a state of “transcriptional chaos.” Conversely, GL treatment restored the transcriptomic profile to a near-healthy baseline level. KEGG analysis highlighted the modulation of the Toll-like receptor (TLR) signaling pathway and arachidonic acid metabolism in the GL group. We propose that *Glycyrrhiza* acts as a “regulator” of the TLR-*NF-κB1* axis (35). Our qRT-PCR data revealed a biphasic immune response: GL initially “primes” the acute response (upregulating *IL-1β* and *TNF-α* at 1 dpi) to combat infection, and most importantly, it subsequently induces a massive upregulation of *IL-10* by 3–7 dpi (**Figure 8**). *IL-10* is a master anti-inflammatory cytokine that is essential for limiting tissue damage. The robust correlation between elevated *IL-10* mRNA levels and reduced gill lamellar fusion (**Figure 5G**) suggests that GL treatment actively triggers the “resolution phase” of inflammation. Furthermore, the enrichment of arachidonic acid metabolism suggests that GL may modulate the class switching of lipid mediators from pro-inflammatory leukotrienes to pro-resolving lipoxins, a mechanism documented in mammalian models but rarely demonstrated in fish (36).

For scaleless, benthic fish, such as *T. yarkandensis*, the integument and gut mucosa are the primary lines of defense against sediment-dwelling pathogens. The histopathological preservation of the gill and hindgut in the GL group is of profound ecological significance (37–39). The prevention of gill lamellar fusion ensures adequate oxygenation in a host that is already stressed by saline-alkaline osmoregulation. Simultaneously, the maintenance of intestinal villi integrity (**Figure 5K**) prevents the “leaky gut” phenomenon, blocking the translocation of bacteria and endotoxins (LPS) into the systemic circulation. This mucosal protection breaks the vicious cycle of reinfection and systemic toxicity, likely contributing to the sustained survival observed (40, 41).

Despite the promising findings, several limitations should be acknowledged. A limitation of this study is the lack of detailed chemical characterization of the *Glycyrrhiza* extract (e.g., HPLC fingerprinting or quantification of key marker compounds such as glycyrrhizic acid). Future studies will incorporate standardized analytical approaches to establish quality control criteria and to link specific active components with therapeutic efficacy.

In summary, this study validated *Glycyrrhiza* extract as an effective therapeutic agent against *S. putrefaciens* infection in *T. yarkandensis*. Its efficacy stems from a synergistic mechanism (1): direct limitation of bacterial colonization (2), attenuation of oxidative stress and tissue edema, and (3) sophisticated modulation of the immune response characterized by the timely upregulation of *IL-10* and regulation of TLR signaling (42). By shifting the host response from an “uncontrolled cytokine storm” to “controlled resolution,” *Glycyrrhiza* offers a sustainable and eco-friendly alternative to antibiotics for disease management in extreme aquaculture environments. Future studies should focus on isolating specific bioactive saponins and optimizing dietary delivery formulations for field applications.

## 5 Conclusion

This study identified *S. putrefaciens* (strain LHL-1) as the etiological agent of hemorrhagic septicemia in indigenous *T. yarkandensis* and elucidated the therapeutic mechanism of *Glycyrrhiza* extract. We found that 20 mg/mL *Glycyrrhiza* extract is a potent non-antibiotic therapeutic agent, achieving a 58.1% survival rate via a dual mechanism. Phenotypically, it reduced bacterial load, ameliorated aberrant hematological and serum biochemical indices, alleviated lethal tissue edema, and protected the gill and intestinal mucosal barriers essential for *T. yarkandensis* in saline-alkaline environments. Mechanistically, transcriptomic analysis revealed that the extract orchestrates systemic inflammation resolution by restoring transcriptomic homeostasis through regulating core immune signaling and arachidonic acid metabolism, and robustly upregulating anti-inflammatory cytokines. This pro-resolution immunomodulation effectively prevents cytokine storm-induced organ failure in patients with untreated infections. Thus, *Glycyrrhiza* extract is a scientifically validated, eco-friendly antibiotic alternative for bacterial diseases in saline-alkaline aquaculture, with its local sourcing in northwest China enhancing its practical application in the Tarim River Basin. In large-scale saline-alkaline aquaculture of *T. yarkandensis*, oral administration of *Glycyrrhiza* extract (GL) through medicated feed represents the most practical and cost-effective delivery route, which enables sustained drug release and acts directly on the intestinal mucosa to strengthen its barrier function. For acute outbreaks of hemorrhagic septicemia, short-term immersion treatment can be used as an emergency supplementary measure, thus forming an integrated strategy of “daily oral prevention combined with emergency immersion therapy”.

## Data Availability

The raw transcriptome sequencing data reported in this study have been deposited in the NCBI Sequence Read Archive (SRA) under the BioProject accession number PRJNA1406777.

## References

[1] Wang X Chen S Li Y Zhang H Cui D Liu Y . Transcriptomic study of muscle in response to heat in Triplophysa yarkandensis. J Fish Biol. (2026) 108(1):190–206. doi: 10.1111/jfb.70184 40967904

[2] Wang Z Hao Y Chen Y Ji Q Ai T Zhang S . Histological study on digestive system of Triplophysa yarkandensis in saline-alkali and freshwater environments: Adaptive mechanisms. Biology. (2025) 14:1187. doi: 10.3390/biology14091187 41007332 PMC12467716

[3] Yang L Yu Y Wang C Hu L Ma Y Zi F . Analysis and evaluation of the muscle quality of high-salinity aquaculture fish in Xinjiang, China. J Food Compos Anal. (2025) 144:107750. doi: 10.1016/j.jfca.2025.107750 38826717

[4] Chen N Jiang J Gao X Li X Zhang Y Liu X . Histopathological analysis and the immune related gene expression profiles of mandarin fish (Siniperca chuatsi) infected with Aeromonas hydrophila. Fish Shellfish Immunol. (2018) 83:410–5. doi: 10.1016/j.fsi.2018.09.023 30201448

[5] Aydin A Sudagidan M Mamatova Z Yurt MNZ Ozalp VC Zornu J . Bacterial skin microbiota of seabass from Aegean fish farms and antibiotic susceptibility of psychrotrophic Pseudomonas. Foods. (2023) 12:1956. doi: 10.3390/foods12101956 37238774 PMC10216923

[6] Gao X Sun M Li B Xie J . The changes of phenotypic characteristics of Shewanella putrefaciens under cold adaptation. J Food Qual. (2023) 2023:9917097. doi: 10.1155/2023/9917097

[7] Zhang X Wang T Zhai D Liu H Xiong F Wang Y . Transcriptome analysis and gene expression analysis related to salinity-alkalinity and low temperature adaptation of Triplophysa yarkandensis. Front Genet. (2022) 13:1089274. doi: 10.3389/fgene.2022.1089274 36712878 PMC9877283

[8] Ouyang H Deng N Xu J Huang J Han C Liu D . Effects of hyperosmotic stress on the intestinal microbiota, transcriptome, and immune function of mandarin fish (Siniperca chuatsi). Aquaculture. (2023) 563:738901. doi: 10.1016/j.aquaculture.2022.738901 38826717

[9] Wang L Yang R Yuan B Liu Y Liu C . The antiviral and antimicrobial activities of licorice, a widely-used Chinese herb. Acta Pharm Sin B. (2015) 5:310–5. doi: 10.1016/j.apsb.2015.05.005 26579460 PMC4629407

[10] Yang R Yuan B Ma Y Wang L Liu C Liu Y . Hmgr, Sqs, B-as, and cytochrome P450 monooxygenase genes in Glycyrrhiza uralensis. Chin Herb Med. (2015) 7:290–5. doi: 10.1016/S1674-6384(15)60054-5

[11] Li T Hua S Ma J Dong L Xu F Fu X . Spectrum-effect relationships of flavonoids in Glycyrrhiza uralensis Fisch. J Anal Methods Chem. (2020) 2020:8838290. doi: 10.1155/2020/8838290 33343964 PMC7728469

[12] Chen X Mai K Zhang W Wang X Ai Q Xu W . Effects of dietary glycyrrhizin on growth and nonspecific immunity of white shrimp, Litopenaeus vannamei. J World Aquacult Soc. (2010) 41:665–74. doi: 10.1111/j.1749-7345.2010.00409.x 40046247

[13] Yang R Zhao W Wang Y Fu Z Hu J Zhou S . Effect of licorice on gene expression related to the growth of Asian seabass Lates calcarifer. J Mar Sci Eng. (2024) 12:1036. doi: 10.3390/jmse12071036 30654563

[14] Yang R Han M Fu Z Wang Y Zhao W Yu G . Immune responses of Asian seabass Lates calcarifer to dietary Glycyrrhiza uralensis. Anim (Basel). (2020) 10:1629. doi: 10.3390/ani10091629 32932808 PMC7552140

[15] Dong F Sun Y-L Qian Y-X Chen Q He J-L Wang J-T . Integrated analysis of transcriptome and metabolome reveals the regulatory mechanism of largemouth bass (Micropterus salmoides) in response to Nocardia seriolae infection. Fish Shellfish Immunol. (2024) 145:109322. doi: 10.1016/j.fsi.2023.109322 38128679

[16] Chen H Kang H Liu Y Ye X Chen Y Ren D . Synergistic efficacy of astragalus polysaccharides and chitosan in treating Aeromonas veronii infections in saline-alkaline water-cultured Nile tilapia (Oreochromis niloticus): Integrating early bactericidal and long-term immunoregulatory effects. Fish Shellfish Immunol. (2026) 168:110911. doi: 10.1016/j.fsi.2025.110911 41062006

[17] Zhou F Chang M Ruan S Huang W Sha Z Cai B . Transcriptomic and histologic analyses preliminarily reveal the immune-metabolic response mechanism to saline-alkaline in large yellow croaker (Larimichthys crocea). Comp Biochem Physiol Part D Genomics Proteomics. (2024) 52:101282. doi: 10.1016/j.cbd.2024.101282 38943980

[18] Ahmadifar E Pourmohammadi Fallah H Yousefi M Dawood MAO Hoseinifar SH Adineh H . The gene regulatory roles of herbal extracts on the growth, immune system, and reproduction of fish. Anim (Basel). (2021) 11:2167. doi: 10.3390/ani11082167 34438625 PMC8388479

[19] Zhang W Zhao J Ma Y Li J Chen X . The effective components of herbal medicines used for prevention and control of fish diseases. Fish Shellfish Immunol. (2022) 126:73–83. doi: 10.1016/j.fsi.2022.05.036 35609759

[20] de Ocenda V-R Almeida-Prieto S Luzardo-Álvarez A Barja JL Otero-Espinar FJ Blanco-Méndez J . Pharmacokinetic model of florfenicol in turbot (Scophthalmus maximus): Establishment of optimal dosage and administration in medicated feed. J Fish Dis. (2017) 40:411–24. doi: 10.1111/jfd.12525 27502011

[21] Wang W Fang P Lin X Xia L Qi C Wang J . Effect of liquorice extracts on the resistance of Carassius auratusto stress and pathogen in fection. Freshw Fisheries. (2007) (4):3–6.

[22] Li X Ju T Zhang Y . Research progress on extraction methods and applications of glycyrrhizic acid. J Lanzhou Jiaotong Univ. (2026) 45(1):161–8. doi: 10.3969/j.issn.2096-9066.2026.01.016

[23] Kuzucu O Özcan M . Molecular characterization of Shewanella putrefaciens and isolation and morphological characterization of its lytic phage. Rev Científica la Facultad Cienc Veterinarias. (2025) 35:7. doi: 10.52973/rcfcv-e35537

[24] Ronish LA Sidner B Yu Y Piepenbrink KH . Recognition of extracellular DNA by type IV pili promotes biofilm formation by Clostridioides difficile. J Biol Chem. (2022) 298:102449. doi: 10.1016/j.jbc.2022.102449 36064001 PMC9556784

[25] Conrad JC . Physics of bacterial near-surface motility using flagella and type IV pili: Implications for biofilm formation. Res Microbiol. (2012) 163:619–29. doi: 10.1016/j.resmic.2012.10.016 23103335

[26] Fonseca DR Chitas R Parreira P Martins MCL . How to manage Helicobacter pylori infection beyond antibiotics: The bioengineering quest. Appl Mater Today. (2024) 37:102123. doi: 10.1016/j.apmt.2024.102123 38826717

[27] Darvishi M Shamsaie Mehrgan M Khajehrahimi AE . Effect of licorice (Glycyrrhiza glabra) extract as an immunostimulant on serum and skin mucus immune parameters, transcriptomic responses of immune-related gene, and disease resistance against Yersinia ruckeri in rainbow trout (Oncorhynchus mykiss). Front Vet Sci. (2022) 9:811684. doi: 10.3389/fvets.2022.811684 35280148 PMC8904569

[28] Tao F Cao J-F Liu Y-J Chen R-Y Shi J-J Li C-Y . Evaluation on the antibacterial activity of glycyrrhizin against Pseudomonas plecoglossicida in ayu fish (Plecoglossus altivelis). Aquaculture. (2025) 595:741520. doi: 10.1016/j.aquaculture.2024.741520 38826717

[29] Xu X Gong L Wang B Wu Y Wang Y Mei X . Glycyrrhizin attenuates Salmonella enterica serovar Typhimurium infection: New insights into its protective mechanism. Front Immunol. (2018) 9:2321. doi: 10.3389/fimmu.2018.02321 30459751 PMC6232675

[30] Reda RM El-Murr A Abdel-Basset NA Metwally MMM Ibrahim RE . Implications of ammonia stress for the pathogenicity of Shewanella spp. in Oreochromis niloticus: Effects on hematological, biochemical, immunological, and histopathological parameters. BMC Vet Res. (2024) 20:324. doi: 10.1186/s12917-024-04175-9 39026304 PMC11256577

[31] Bode C Weis S Sauer A Wendel-Garcia P David S . Targeting the host response in sepsis: Current approaches and future evidence. Crit Care. (2023) 27:478. doi: 10.1186/s13054-023-04762-6 38057824 PMC10698949

[32] Huang M Cai S Su J . The pathogenesis of sepsis and potential therapeutic targets. Int J Mol Sci. (2019) 20:5376. doi: 10.3390/ijms20215376 31671729 PMC6862039

[33] Lu L-H Chao C-H Yeh T-M . Inhibition of autophagy protects against sepsis by concurrently attenuating the cytokine storm and vascular leakage. J Infect. (2019) 78:178–86. doi: 10.1016/j.jinf.2018.12.003 30653985

[34] Zhu W Zhang Y Liao Z Huo X Yang C Zhang Y . Ifn1 enhances thrombocyte phagocytosis through Ifn receptor complex-Jak/Stat-complement C3.3-Cr1 pathway and facilitates antibacterial immune regulation in teleost. J Immunol. (2023) 210:1043–58. doi: 10.4049/jimmunol.2200787 36883974

[35] Kong D Wang Z Tian J Liu T Zhou H . Glycyrrhizin inactivates toll-like receptor (Tlr) signaling pathway to reduce lipopolysaccharide-induced acute lung injury by inhibiting Tlr2. J Cell Physiol. (2019) 234:4597–607. doi: 10.1002/jcp.27242 30203548

[36] Bottiglione F Dee CT Lea R Zeef LAH Badrock AP Wane M . Zebrafish Il-4-like cytokines and Il-10 suppress inflammation but only Il-10 is essential for gill homeostasis. J Immunol. (2020) 205:994–1008. doi: 10.4049/jimmunol.2000372 32641385 PMC7416321

[37] Ding L Li W Liang L Huang Z Li N Zhang J . Modulation of the intestinal barrier adaptive functions in red-eared slider (Trachemys scripta elegans) invading brackish waters. Sci Total Environ. (2021) 751:141744. doi: 10.1016/j.scitotenv.2020.141744 32890802

[38] Ni L Jin L Zeng M Xu Y Wang Y Peng Z . The cavefish Triplophysa rosa has a well-developed adaptive immune system: Evidence from histological and comparative genomic analysis. Aquaculture. (2024) 581:740395. doi: 10.1016/j.aquaculture.2023.740395 38826717

[39] Horn V Sonnenberg GF . Group 3 innate lymphoid cells in intestinal health and disease. Nat Rev Gastroenterol Hepatol. (2024) 21:428–43. doi: 10.1038/s41575-024-00906-3 38467885 PMC11144103

[40] Costa PM Diniz MS Caeiro S Lobo J Martins M Ferreira AM . Histological biomarkers in liver and gills of juvenile Solea Senegalensis exposed to contaminated estuarine sediments: A weighted indices approach. Aquat Toxicol. (2009) 92:202–12. doi: 10.1016/j.aquatox.2008.12.009 19282038

[41] Rodrigues S Antunes SC Nunes B Correia AT . Histopathological effects of the antibiotic erythromycin on the freshwater fish species Oncorhynchus mykiss. Ecotoxicology Environ Saf. (2019) 181:1–10. doi: 10.1016/j.ecoenv.2019.05.067 31154114

[42] Su J . Toll-like receptor signaling in teleosts. Sci China Life Sci. (2025) 68:1889–911. doi: 10.1007/s11427-024-2822-5 39961973

