# Supplementary material for: Glycyrrhiza extract alleviates hemorrhagic septicemia in Triplophysa yarkandensis infected with Shewanella putrefaciens: integrated bactericidal and immunomodulatory effects
Source: Front Immunol. 2026 Jun 26;17:1839757. doi: 10.3389/fimmu.2026.1839757 (PMC13349888; doi:10.3389/fimmu.2026.1839757)
Supplement: Supplementary file 1 [file SupplementaryFile1.docx]

Supplementary Material

## Supplementary Figures


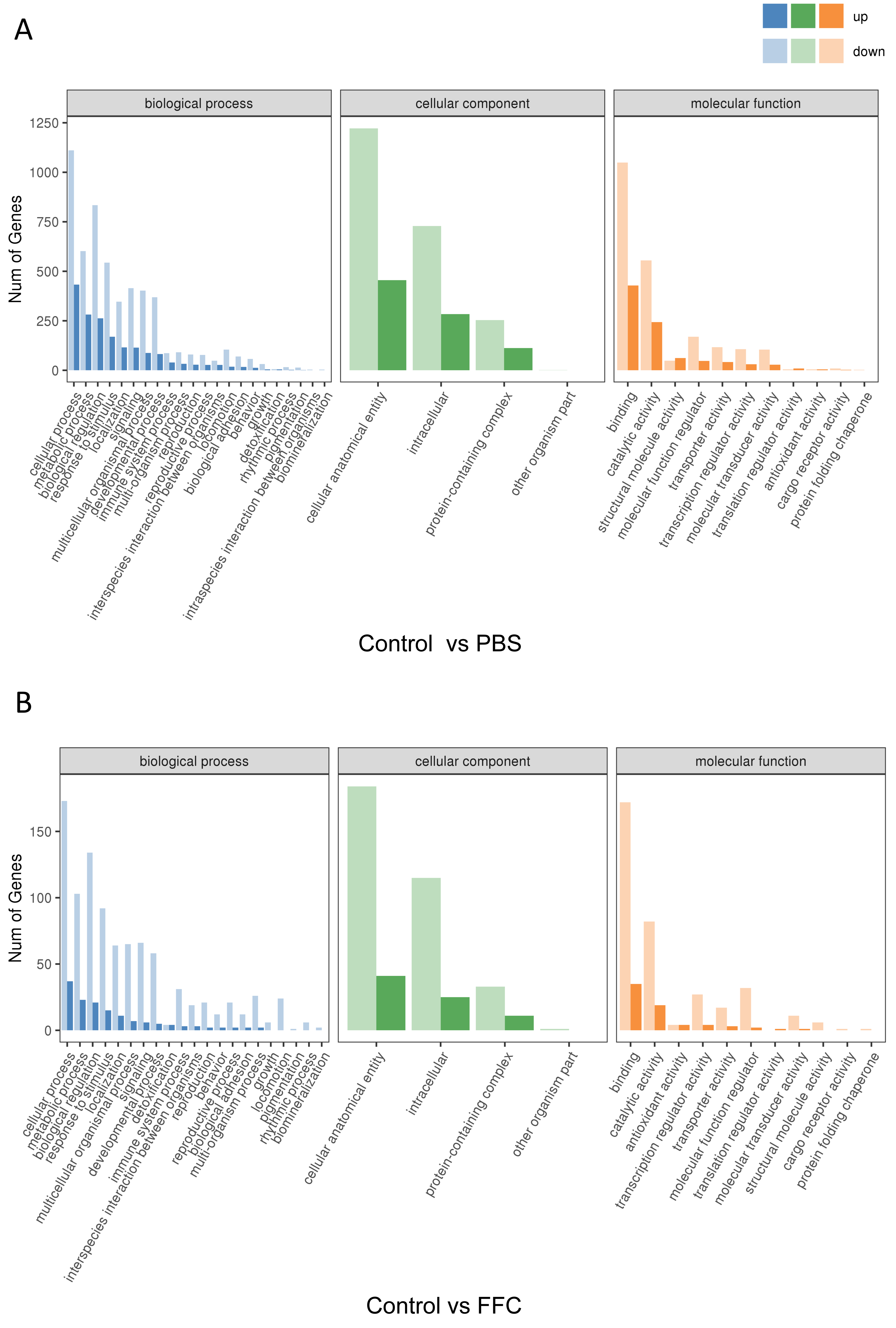


**Supplementary Figure 1.** GO classification of (DEGs) in the Untreated and Antibiotic-treated groups.The histograms display the count of up-regulated (dark color) and down-regulated (light color) genes assigned to level-2 GO terms in Biological Process, Cellular Component, and Molecular Function. **(A)** Control vs PBS (Untreated Infection): Demonstrates a massive number of DEGs (y-axis > 1250), reflecting profound systemic physiological perturbation and metabolic dysregulation caused by the S. putrefaciens infection. **(B)** Control vs FFC (Antibiotic Treatment): Shows a reduced number of DEGs (y-axis < 175) compared to the PBS group, indicating that antibiotic treatment significantly attenuates transcriptional stress, though residual metabolic upregulation persists.

**
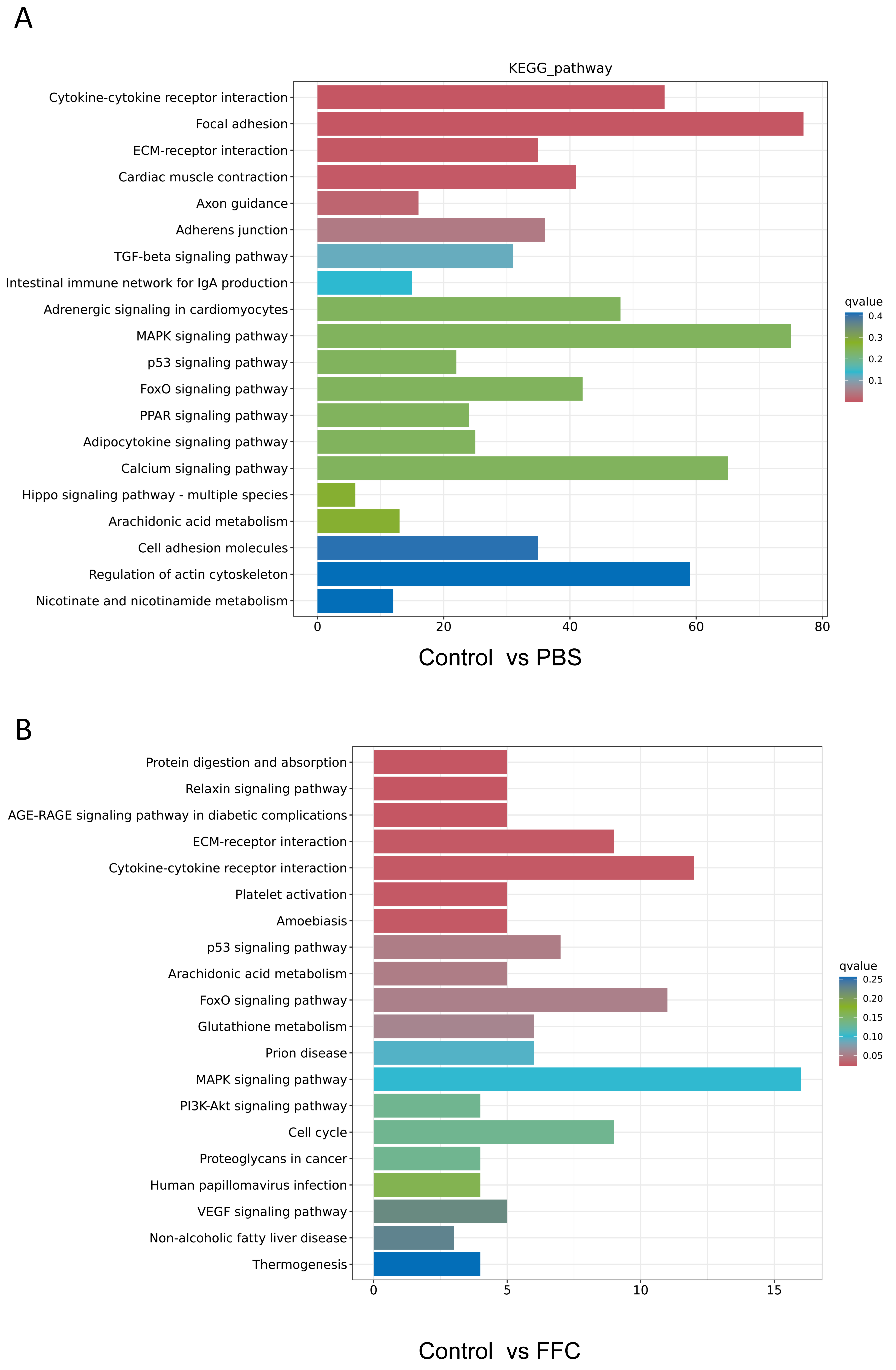
**

**Supplementary Figure 2.** KEGG Pathway Enrichment Analysis of DEGs in the Untreated and Antibiotic-treated groups. Top 20 enriched signaling pathways based on Q-value (color scale) and gene count (bar height). **(A)** Control vs PBS: The pathway profile is dominated by "Focal adhesion," "ECM-receptor interaction," and "Cytokine-cytokine receptor interaction." This signifies severe immunopathology, characterized by a cytokine storm and the breakdown of structural tissue integrity (correlating with the high tissue edema observed). **(B)** Control vs FFC: The profile shifts towards "MAPK signaling," "FoxO signaling," and "PI3K-Akt signaling," representing general cellular survival and stress response pathways during recovery. Notably, the inflammatory "Cytokine-cytokine" pathway is significantly reduced compared to the PBS group.
